# Quinolone antibiotics

**DOI:** 10.1039/c9md00120d

**Published:** 2019-06-28

**Authors:** Thu D. M. Pham, Zyta M. Ziora, Mark A. T. Blaskovich

**Affiliations:** a School of Chemistry & Molecular Biosciences , The University of Queensland , Brisbane , QLD 4072 , Australia; b Institute for Molecular Bioscience , The University of Queensland , Brisbane , QLD 4072 , Australia . Email: m.blaskovich@uq.edu.au

## Abstract

The quinolone antibiotics arose in the early 1960s, with the first examples possessing a narrow-spectrum of activity with unfavorable pharmacokinetic properties. Over time, the development of new quinolone antibiotics has led to improved analogues with an expanded spectrum and high efficacy. Nowadays, quinolones are widely used for treating a variety of infections. Quinolones are broad-spectrum antibiotics that are active against both Gram-positive and Gram-negative bacteria, including mycobacteria, and anaerobes. They exert their actions by inhibiting bacterial nucleic acid synthesis through disrupting the enzymes topoisomerase IV and DNA gyrase, and by causing breakage of bacterial chromosomes. However, bacteria have acquired resistance to quinolones, similar to other antibacterial agents, due to the overuse of these drugs. Mechanisms contributing to quinolone resistance are mediated by chromosomal mutations and/or plasmid gene uptake that alter the topoisomerase targets, modify the quinolone, and/or reduce drug accumulation by either decreased uptake or increased efflux. This review discusses the development of this class of antibiotics in terms of potency, pharmacokinetics and toxicity, along with the resistance mechanisms which reduce the quinolones' activity against pathogens. Potential strategies for future generations of quinolone antibiotics with enhanced activity against resistant strains are suggested.

## Abstract 2

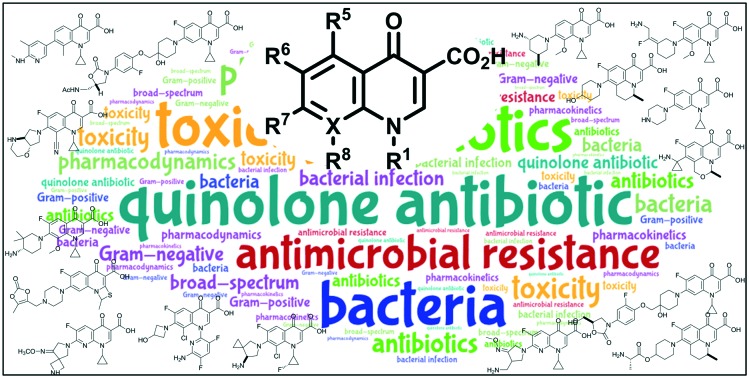
The quinolone antibiotics arose in the early 1960s, with the first examples possessing a narrow-spectrum activity with unfavorable pharmacokinetic properties.

## 1. Introduction

**Fig. 1:**
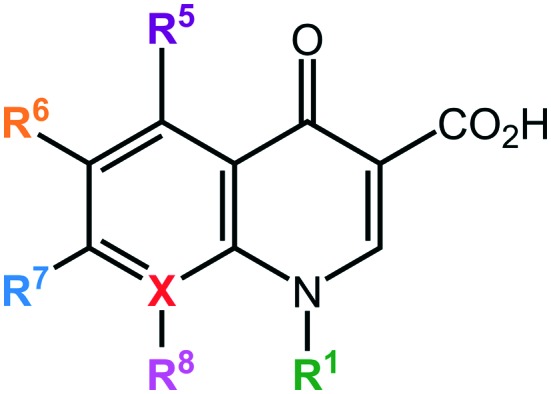
Core structure of quinolone antibiotics. There are 6 important positions for modifications to improve the activity of the drug: R_1_, R_5_, R_6_, R_7_, R_8_, and X. X = C defines quinolones, X = N defines naphthyridones.

The quinolones are a family of antibiotics containing a bicyclic core structure related to the compound 4-quinolone (Fig. 1).^1^ Since their discovery in the early 1960s, they have gained increasing importance as key therapies to treat both community-acquired and severe hospital-acquired infections.^2^ The first quinolone antibiotic is generally considered to be nalidixic acid, which was reported in 1962 as part of a series of 1-alkyl-1,8-naphthyridines prepared at the Sterling-Winthrop Research Institute.^3^ However, a 2015 perspective that examined the origins of quinolone antibiotics in greater detail points out that the author of the 1962 publication (George Lesher) described the isolation of-chloro-1-ethyl-1,4-dihydro-4-oxo-3-quinolinecarboxylic acid in the late 1950s as a by-product of chloroquine synthesis, with modest antibacterial activity leading to further work on analogues, including nalidixic acid.^1^ Around the same time, Imperial Chemical Industries (ICI) published patent applications with antibacterial quinolones, including a 6-fluoroquinolone.^1^ Nalidixic acid is a narrow-spectrum agent against enteric bacteria used for treating uncomplicated urinary tract infections (UTIs).^4^ During the 1970s–1980s, the coverage of the quinolone class was expanded significantly by the breakthrough development of fluoroquinolones, which show a much broader spectrum of activity and improved pharmacokinetics compared to the first-generation quinolone.^5^ Those fluoroquinolones, such as ciprofloxacin and ofloxacin, are active against both Gram-negative and Gram-positive pathogens; importantly, they are also active against the causative agent of tuberculosis, *Mycobacterium tuberculosis*. Quinolones have been favoured as antibiotics for more than five decades because of their high potency, broad spectrum of activity, favorable bioavailability, convenient formulations, and high serum concentrations, as well as a comparatively low incidence of side effects.^6^ Quinolones are widely prescribed for several different types of human infections,^7^ with side effects including gastrointestinal reactions, CNS reactions, genotoxicity, phototoxicity, and some minor adverse effects.

The quinolone class of antibiotics inhibits the DNA synthesis of bacteria by disrupting the bacterial topoisomerase type II; inhibiting the catalytic activity of DNA gyrase and topoisomerase IV.^8^ These two enzymes are critical bacterial enzymes that regulate the chromosomal supercoiling required for DNA synthesis.^9^ Over time, quinolone resistance has become a serious problem among many emerging resistant pathogens.^10^ The mutations generated by the bacteria against quinolones are generally located on the target enzyme binding sites in DNA gyrase and topoisomerase IV.^11^ In addition, resistance to this class of antibiotics can be obtained by acquisition of a resistant plasmid from other sources in the environment through horizontal transfer, leading to the rapid spread of resistance.^12^

This review discusses the current knowledge of the development process of quinolones on how structural modifications in the evolving generations have mediated improvements in terms of potency, pharmacokinetics, and toxicity. It also summarizes the relevant knowledge of mode of actions and resistance. Lastly, the review examines future strategies to improve the activity of this class and overcome the resistance.

## 2. Development of the quinolones

The prototypical quinolone, nalidixic acid (technically a naphthyridone), was discovered in the 1960s as a by-product during the synthesis of anti-malarial quinine compounds.^3^ It was soon found to act by inhibiting the activity of bacterial topoisomerase type II enzymes, inhibiting the bacterial replication.^13^ In 1967, nalidixic acid was approved for clinical treatment for urinary tract infections (UTIs) caused by Gram-negative bacteria.^4^ However, its use was limited because of the narrow spectrum of activity, low serum concentrations achieved, high inhibitory concentration required, and several adverse effects.^4^ It was not until the 1980s that improved analogues were made, when the need for new treatments of diarrhea and UTIs caused by resistant *Shigella* and *Escherichia coli* led the attention of researchers to improve the activity and optimize the toxicity of the quinolones.

Many researchers have studied the structure–activity relationships of quinolone antibiotics. Fig. 1 presents the core structure of the basic quinolones with two major groups developed from it: quinolones and naphthyridones, which can be identified by the ‘X’ position. A carbon atom at the X position defines the quinolones, while a nitrogen atom at the X position defines the naphthyridones.^14^ Based on their spectrum of activity, quinolones are classified into four generations.^15^ The development of quinolones from generation to generation to obtain broader spectrum activity has proceeded by addition of different substituents into different position on the pharmacophore. Table 1 presents a summary of the quinolone development process.

**Table 1:** Overview of the development of quinolone antibiotic generations. Quinolone antibiotics develop from generations to generations to obtain broader activity spectrum by the addition of different substituents into different positions to the core structure

Generation	Name	Structure	Antimicrobial spectrum	Modifications	Comment
1	Nalidixic acid	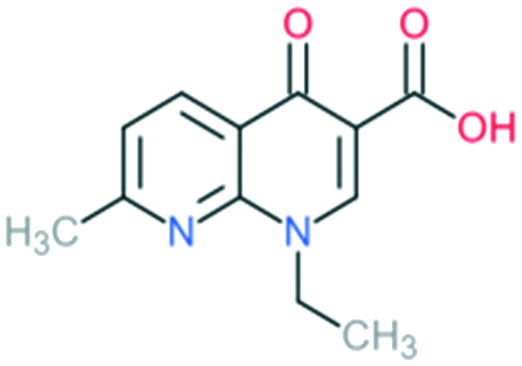	Gram-negative organisms (except *Pseudomonas* species)	N at X_8_ position = naphthyridone	First molecule to be discovered in quinolone class
2a	Enoxacin	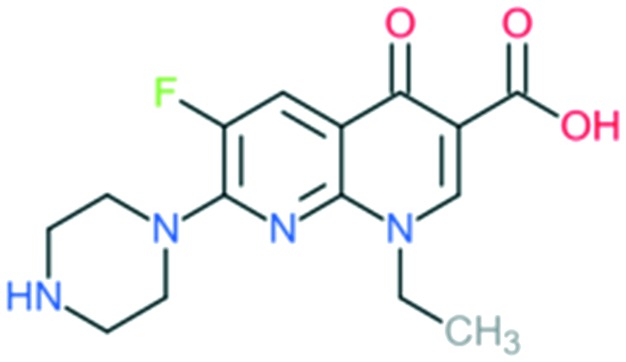	All Gram-negative pathogens and some atypical pathogens (including *Mycoplasma pneumoniae* and *Chlamydia pneumoniae*)	Addition of (1) piperazine to C_7_ position, and (2) –F to C_6_ position	(1) Improves activity against Gram-negative organisms (inhibits the efflux mechanism)
	Norfloxacin	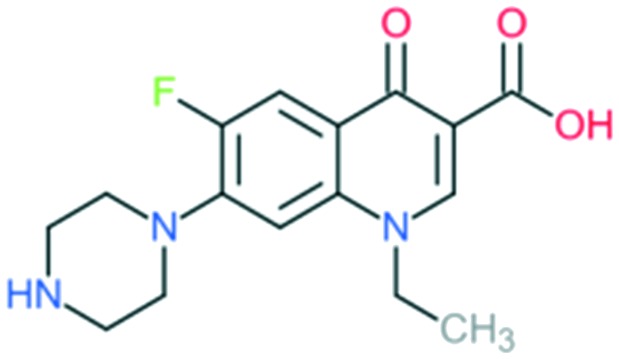		Addition of (1) piperazine to C_7_ position (quinolone), and (2) –F to C_6_ position	(1) Improves bioavailability, side effects
					Improves activity against Gram-negative organisms (inhibits the efflux mechanism)
	Ciprofloxacin	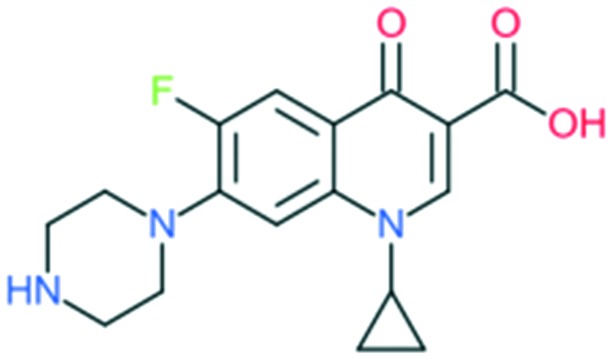		Addition of (1) piperazine to C_7_ position, (2) –F to C_6_ position, and (3) cyclopropyl at the N_1_ position	(1) Improves anti-Gram-negative activity
					(2) Increases potency
2b	Ofloxacin (l-isomer = levofloxacin)	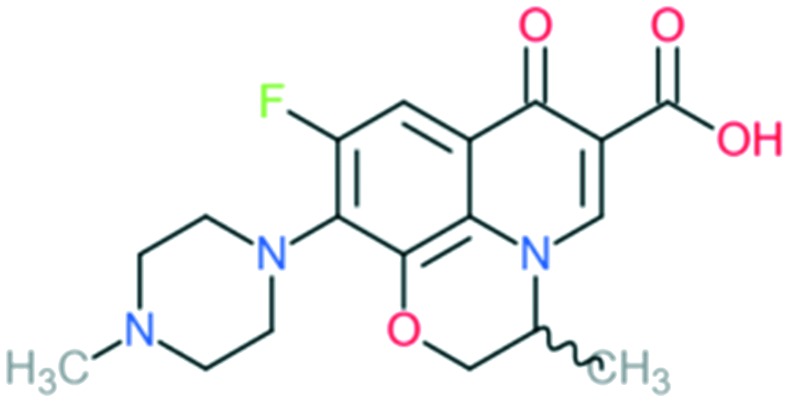	All Gram-negative pathogens and some Gram-positive bacteria (including *Staphylococcus aureus*, except *Streptococcus pneumoniae*) and some atypical organisms	Addition of (1) methylated piperazine to C_7_ position and (2) –OCH_2_ at C_8_ position	(1) Increases anti-Gram-positive activity
					(2) Increases anti-Gram-positive activity, tissue penetration, half-life
					(3) l-Isomer is 4-fold more active
	Lomefloxacin	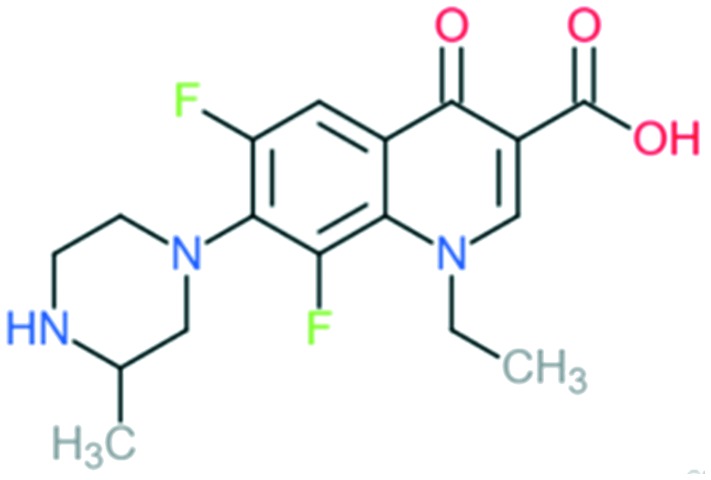		Addition of (1) methylated piperazine to C_7_ position and (2) –F at C_8_ position	(1) Increases anti-Gram-positive activity
					(2) Increases anti-Gram-positive activity, tissue penetration, half-life
3	Sparfloxacin	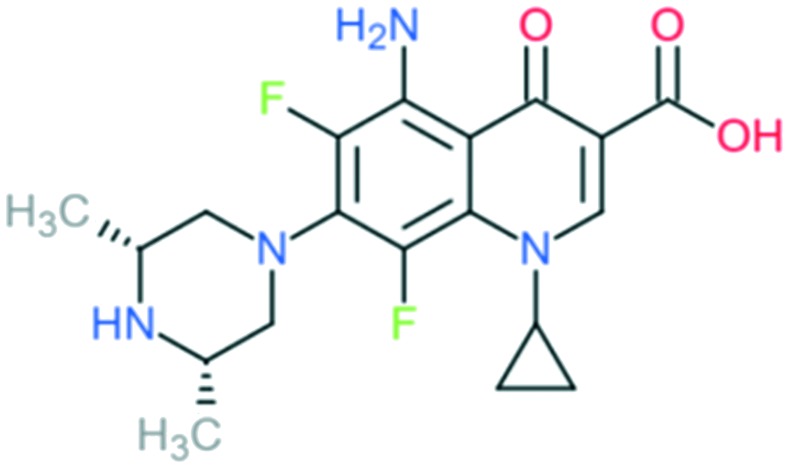	Retains the activity of second-generation drugs and possesses expanded Gram-positive coverage (penicillin-sensitive and penicillin-resistant *S. pneumoniae*) and improved activity against atypical pathogens	Addition of (1) dimethylated piperazine to C_7_ position, (2) –F at C_6_ and C_8_ positions, (3) –NH_2_ at C_5_ position, and (4) cyclopropyl ring at N_1_ position	(1) Increases anti-Gram-positive activity
					(2) Increases anti-Gram-positive activity, tissue penetration, half-life
					(3) Improves activity against Gram-positive pathogens
					(4) Improves potency of the drug
	Grepafloxacin	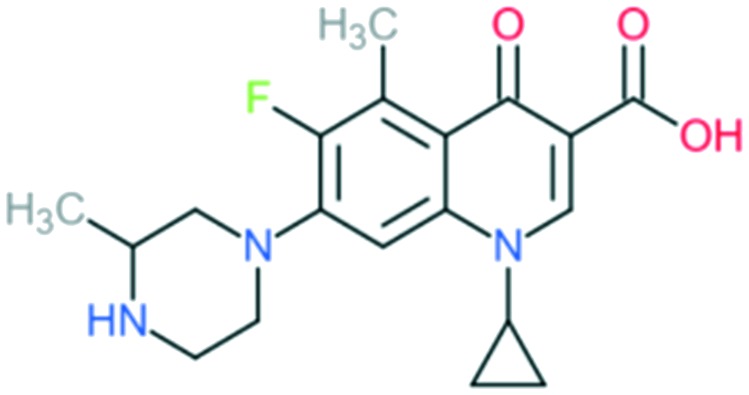		Addition of (1) methylated piperazine to C_7_ position, (2) –CH_3_ at C_5_ position, and (3) cyclopropyl ring at N_1_ position	(1) Improves anti-Gram-positive activity
					(2) Improves anti-Gram-positive activity compared to ciprofloxacin
					(3) Improves potency of the drug
	Clinafloxacin	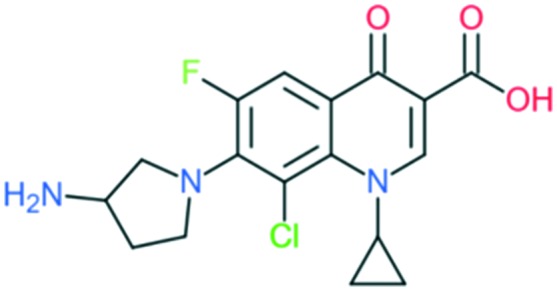		Addition of (1) 3-aminopyrrolidin-1-yl group to C_7_ position, (2) –Cl at C_8_ position, and (3) cyclopropyl ring at N_1_ position	(1) Improves anti-Gram-positive activity and overcomes physical disadvantages
					(2) Improve anti-Gram-positive activity, tissue penetration, half-life
					(3) Improves potency of the drug
	Gatifloxacin	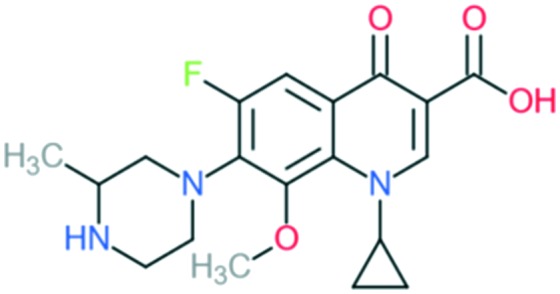		Addition of (1) methylated piperazine group to C_7_ position, (2) methoxy group at C_8_ position, and (3) cyclopropyl ring at N_1_ position	(1) Improves anti-Gram-positive activity
					(2) Improves anti-Gram-positive activity, tissue penetration, half-life
					(3) Improves potency of the drug
4	Moxifloxacin	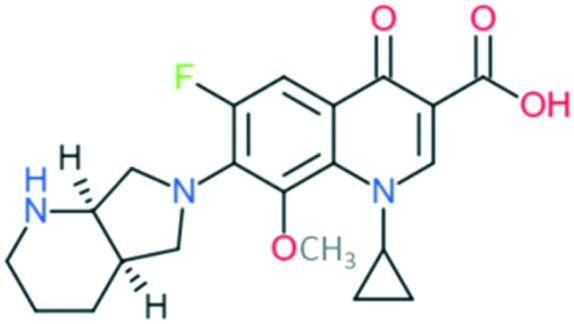	Covers all the activities of third generation drugs and extra anaerobic activity	Addition of (1) azabicyclo group to C_7_ position, (2) –OCH_3_ at C_8_ position, and (3) cyclopropyl ring at N_1_ position	(1) Improves anti-Gram-positive activity but may result in low water solubility and oral bioavailability
					(2) Improves anti-Gram-positive activity, tissue penetration, half-life
					(3) Improves potency of the drug
	Gemifloxacin	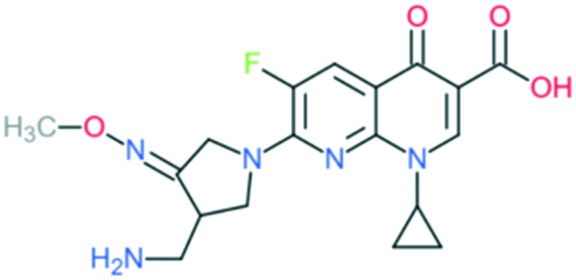		Addition of (1) 3-methoximine-4-aminomethyl-pyrrolidin-1-yl group to C_7_ position and (2) cyclopropyl ring at N_1_ position	(1) Improves anti-Gram-positive activity and overcomes the physical disadvantages compared with pyrrolidine group alone
					(2) Improves potency of the drug
	Trovafloxacin	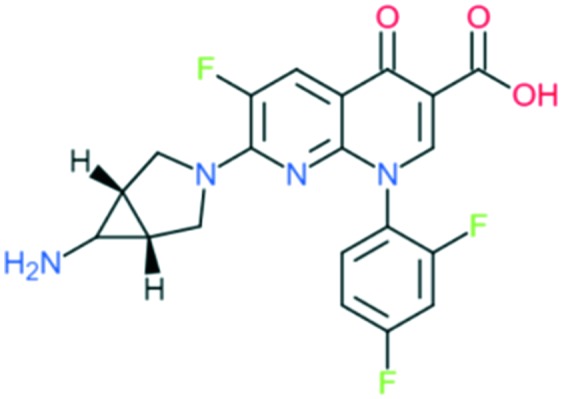		Addition of (1) amine-substituted bicyclic pyrrolidin-1-yl group to C_7_ position and (2) 2,4-difluorophenyl group at N_1_ position	(1) Improve anti-Gram-positive activity
					(2) Improves potency and activity against anaerobes
	Garenoxacin	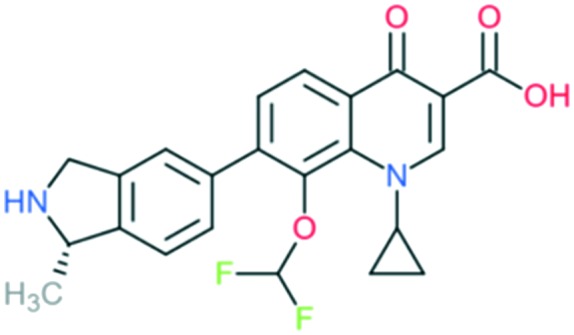		Addition of (1) azabicyclo group to C_7_ position, (2) cyclopropyl group at N_1_, and (3) difluoromethyl ether group at C_8_ position	(1) Significantly improves anti-Gram-positive activity (lipophilicity and half-lives)
					(2) Improves potency of the drug
					(3) Improves anti-Gram-positive activity

### 2.1. Development in activity

The first-generation quinolone activity was limited to only Gram-negative organisms, excluding *Pseudomonas* species.^16^ Shortly after the clinical introduction of nalidixic acid, it was found to cause rapid resistance development in a number of organisms, reducing its effectiveness^17^ and leading to investigations to discover analogues with improved properties.

The first second-generation quinolone, flumequine, exemplified the discovery that a key modification, adding a fluorine (F) atom at the R_6_ position, could significantly improve the spectrum of activity.^18^ This change dramatically increased the quinolone activity, since almost all quinolone antibiotics have been designated as fluoroquinolones, with the exception of the most recent compounds from the fourth generation. Other fluoroquinolones from the second generation include enoxacin, norfloxacin, and ciprofloxacin, which were able to inhibit all Gram-negative organisms, including *Pseudomonas* species.^19^ In addition to the fluoro substituent, these drugs were further modified by addition of a piperazine ring to the R_7_ position and addition of a cyclopropyl group to the R_1_ position. The R_7_ piperazine ring improved the Gram-negative potency,^20^ while the cyclopropyl group was found to improve the overall activity of the compounds.^21^ This combination made ciprofloxacin the most active compound among the early compounds of the second generation and made it the first choice used against *Pseudomonas aeruginosa* today.^22^ Subsequent development of the second generation produced analogues with activity against some Gram-positive bacteria, including *Staphylococcus aureus* but not *Streptococcus pneumoniae*, and some atypical organisms (*Mycoplasma pneumoniae* and *Chlamydia pneumoniae*).^23^ The presence of an alkylated piperazine group at the R_7_ position, as in ofloxacin, marked the first modifications that help inhibit Gram-positive organisms.^24^ The addition of an –OCH_3_ substituent to the R_8_ position of the latter group also helped to improve Gram-positive activity.^25^ Of all compounds in this latter group (2b), ofloxacin is considered as the most powerful as it combines all the new substituents and it is now still being used for clinical treatment. Ofloxacin is a chiral molecule and its l-isomer is the only active compound, which is known as levofloxacin. It was proposed to have 4-fold higher activity compared with ofloxacin and is also more active than ciprofloxacin in treating some strains.^26^,^27^

With the synthesis of fleroxacin, the quinolones entered their third generation. The improvements of this generation included addition of alkylated piperazine and pyrrolidinyl groups to the R_7_ position, and –NH_2_, –OH, and –CH_3_ groups to the R_5_ position to the pharmacophore. The cyclopropyl group at the R_1_ position and the –OCH_3_ group at position R_8_ were kept unchanged from the second generation. The third generation also added new substituents, such as a chloro group (Cl) at the R_8_ position; this was verified to improve the anti-Gram-positive activity of the drug.^25^ Among all modifications at this position, 8-methoxyquinolone was shown to surpass other compounds in activity and spectrum. The improvement is best exemplified by comparing grepafloxacin and gatifloxacin; the MIC_90_ of gatifloxacin (8-MeO) improved significantly compared with that of grepafloxacin (8-H) (Table 2). These modifications expanded the Gram-positive activity of the third generation, including penicillin-sensitive and penicillin-resistant *S. pneumoniae*, while the activity against atypical bacteria was also increased. While a piperazine group in the second generation improved the Gram-negative activity, the alkylated form of this group added to the Gram-positive activity of the fluoroquinolone compounds. A pyrrolidinyl group in this position showed the same improvement as the alkylated piperazine group.^28^ Manipulation of the group at the R_5_ position was shown to increase the activity against Gram-positive organisms.^26^,^29^ The antibacterial potency improvement mediated by substitution at this position was found to increase in the order –CH_3_, –OH, –NH_2_, respectively.^30^ All the modifications (positions R_8_, R_5_, and R_7_) presented in this third generation were designed to improve the activity against Gram-positive bacteria. Among these modifications, manipulation at the R_7_ has generally been the most effective. It can be observed by comparing the MIC_90_ of these compounds. Clinafloxacin is described to possess the most potential among these third-generation drugs, with a methylated pyrrolidinyl group at R_7_ and chlorine at C_8_. The MIC_90_ of clinafloxacin is the lowest in this group (Table 2). There are similarities between the structures of ciprofloxacin and sparfloxacin, but addition of –NH_2_ at R_5_ and alkylation of the piperazine group make the potency of sparfloxacin better than that of ciprofloxacin (Table 2). It is similar in the case of grepafloxacin, with the –CH_3_ substituted.

**Table 2:** Comparative MIC_90_s of quinolones. The potency of the drugs presented in MIC_90_ (mg L^–1^) of each drug on different Gram-negative strains and Gram-positive strains^32^–^49^

MIC_90_ (mg L^–1^)										
	Gram-negative pathogens					Gram-positive pathogens				
	*E. coli*	*P. aeruginosa*	*Klebsiella* spp.	*B. fragilis*	*Haemophilus influenzae*	*S. aureus*	*S. pneumoniae*	Group A *Streptococci*	*Enterococcus* spp.	*Clostridium perfringens*
Nalidixic acid	8	>64	16	>64	2	>64	>64	>64	>64	>64
Enoxacin	0.25	>64	2	>64	0.12	2	64	>64	8	>64
Norfloxacin	0.12	2	0.5	>64	0.06	1	16	4	4	ND
Ciprofloxacin	0.03	1	0.25	16	0.03	1	2	1	4	0.5
Ofloxacin	0.12	4	0.5	16	0.03	0.5	2	2	2	1
Lomefloxacin	0.06	2	0.25	ND	0.06	2	4	4	4	ND
Sparfloxacin	0.06	4	0.5	4	0.03	0.12	0.5	1	2	0.25
Grepafloxacin	0.06	8	0.12	8	0.01	0.12	0.25	1	4	1
Clinafloxacin	0.01	0.5	0.03	0.25	0.01	0.06	0.12	0.06	0.25	0.12
Gatifloxacin	0.06	4	0.25	1	0.03	0.25	0.25	0.25	1	0.5
Moxifloxacin	0.06	8	0.12	1	0.06	0.06	0.12	0.25	2	0.25
Gemifloxacin	0.03	4	0.25	ND	0.06	0.06	0.03	0.06	4	ND
Trovafloxacin	0.06	1	0.25	0.25	0.01	0.03	0.12	0.25	1	0.25
Garenoxacin	0.06	16	0.5	1	0.03	0.03	0.12	0.25	0.5	0.25

The spectrum of activity of fourth-generation compounds covers all the criteria of the third generation with the addition of activity against anaerobic organisms.^23^ The presence of nitrogen (N) at the R_8_ position is responsible for the improved activity against anaerobes,^31^ while a 2,4-difluorophenyl group at the N position improves the overall potency of the drug.^24^ This modification can be seen from the structures of moxifloxacin, gemifloxacin, and trovafloxacin (Table 1). Other modifications are addition of an azabicyclic group and a bulky side chain on the pyrrolidine group at the R_7_ position and addition of a difluoromethyl ether group at the R_8_ position, which all improve the Gram-positive activity.^21^ The azabicyclic group at the R_7_ position produced the highest potency against the Gram-positive bacteria, as demonstrated by comparing the potency and structure between moxifloxacin and gatifloxacin. These two compounds have an otherwise similar structure, differing only at the R_7_ position. The azabicyclic group in moxifloxacin substantially improves Gram-positive potency compared with gatifloxacin (Table 2).

**Fig. 2:**
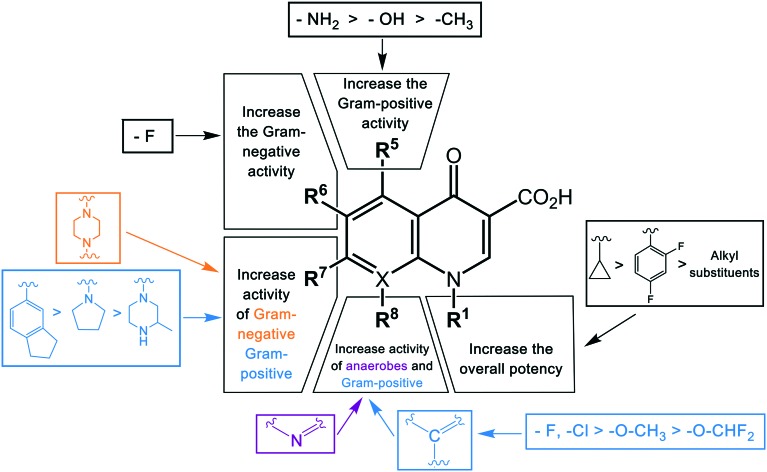
The structure–activity relationships (SAR) of quinolones. The antibacterial activity of quinolones is improved by modifications of different substituents in different positions. The color of the groups in the bracket correlates with the type of activities.

As discussed above, the development of the structure–activity relationship of quinolones through successive generations can be summarized in Fig. 2.

### 2.2. Development in pharmacokinetics (PK) and pharmacodynamics (PD)

The development of quinolones in terms of pharmacokinetics and pharmacodynamics relates to improvements in metabolism, elimination, and transportation, leading to improved antibiotic dosing strategies to enhance the efficacy and prevention of resistant mutations. Use of the very first quinolone agent, nalidixic acid, was limited because it had low serum levels; therefore, it was used as a urinary agent only.^50^ The modifications in the structure of later generations of quinolones led to improved oral absorption as well as larger area under the curve (AUC) and/or maximum serum concentrations (*C*_max_) compared to nalidixic acid.^5^ Those modifications also produced longer elimination half-lives, which permitted once-daily dosing for some agents of the second generation and all agents of later generations (Table 3). Since most of the earlier quinolones had low serum levels and moderate potency, they required frequent doses, with the once-daily dosing of latter agents resulting not only from better exposure but also from their significantly enhanced potency. They also had better tissue penetration.^5^ There is no trend in the extent of protein binding related to the structural modifications. This parameter varies between agents, with some <30% (norfloxacin, lomefloxacin, and gatifloxacin) and others >80% (nalidixic acid, trovafloxacin, and garenoxacin). Over time, changes in the metabolism of quinolones were observed; although earlier quinolones were primarily eliminated by metabolism and renal clearance, later quinolones were modified to become non-renal clearance agents (sparfloxacin, moxifloxacin, gemifloxacin, trovafloxacin, and garenoxacin) (Table 3).

**Table 3:** The development of quinolone antibiotics in pharmacokinetics^33^,^42^,^43^,^51^,^52^

Quinolone	Dose (g) (frequency per day)	*C*_max_ (mg L^–1^)	AUC (mg h L^–1^)	Half-life (h)	Protein binding (%)	Elimination route
Nalidixic acid	1 (×4)	Variable	Variable	1.5	90	Renal
Enoxacin	0.6 (×1)	3.7	29	2	60	Renal
Norfloxacin	0.4 (×2)	1.5	10	3	15	Renal hepatic
Ciprofloxacin	0.75 (×2)	3.5	30	4	40	Renal and enteral
Ofloxacin	0.4 (×2)	4.8	64	6	40	Renal
Lomefloxacin	0.4 (×1)	2.8	26	8	10	Renal
Sparfloxacin	0.4 (×1)	1.0	20	18	40	Renal
Grepafloxacin	0.4 (×1)	1.4	14	14	50	Hepatic
Clinafloxacin	0.2 (×2)	1.6	18	6	40	Renal
Gatifloxacin	0.4 (×1)	3.8	33	12	20	Renal
Moxifloxacin	0.4 (×1)	3.1	30	13	50	Hepatic
Gemifloxacin	0.32 (×1)	1.0	9	7	60	Renal and other
Trovafloxacin	0.3 (×1)	2.5	40	12	85	Hepatic
Garenoxacin	0.4 (×1)	5.8	59	15	87	Renal and other

The quinolones show concentration-dependent killing (CDK) with persistent post-antibiotic effect (PAE),^53^ and the therapeutic outcomes of this group are based on either the AUC/MIC ratio or the *C*_max_/MIC ratio. Clearly, a high AUC or *C*_max_ value combined with low MIC is ideal for increasing the ratio and thereby improving the efficacy. For decades, it was debated as to which ratio best indicated the microbiological and clinical outcomes of quinolones.^30^ It was not until the alarming rise in resistance to ciprofloxacin when treating infections with common low-dose regimens^54^–^56^ that large-scale clinical studies were conducted to define the PD parameters for predicting efficacy. According to several studies, the second-generation quinolones did not obtain a high *C*_max_/MIC ratio,^28^ with the AUC/MIC ratio more accurately reflecting their efficacy. It was shown that an AUC/MIC ratio of >125 indicated the best therapeutic outcomes, and any agents with a *C*_max_/MIC ratio lower than 4 indicated sub-optimal outcomes.^57^,^58^ However, it is still uncertain what the minimum acceptable AUC/MIC ratio is. Some researchers have proposed that an AUC/MIC ratio of 25 is appropriate for use in mild infections and immunocompetent patients, while a value of ≥100 is needed for serious infections and immunocompromised patients.^59^

While the AUC/MIC ratio is used to determine the microbiological outcome of quinolone treatment, the *C*_max_/MIC ratio has been determined to be a factor for preventing the emergence of resistance to quinolones.^30^ A higher *C*_max_ is preferable for lower resistance occurrence. Many *in vitro* studies showed that a low AUC/MIC ratio will increase the selection of resistant mutants, even if this ratio is clinically effective for the infections.^60^–^62^ Combined with the *C*_max_/MIC ratio, a “mutant prevention concentration” (MPC) was developed for prevention of resistance. It is the concentration necessary to prevent the growth of the least susceptible, single-step mutants, with 10^10^ bacteria incubated in the presence of different increasing concentrations of the antibiotics. The MPC is the concentration in which there is no observation of growth of that bacteria.^63^ This MPC is used to prevent resistance during therapy, suggesting a minimum serum concentration to be achieved. This target was used during the development of the third generation of quinolones (gatifloxacin, gemifloxacin, moxifloxacin); they exert lower MPC values than the earlier quinolones when used against *Streptococcus pneumoniae*.^64^,^65^ Accordingly, the MPC of ciprofloxacin for *Pseudomonas aeruginosa* is lower than that of levofloxacin.^66^

**Fig. 3:**
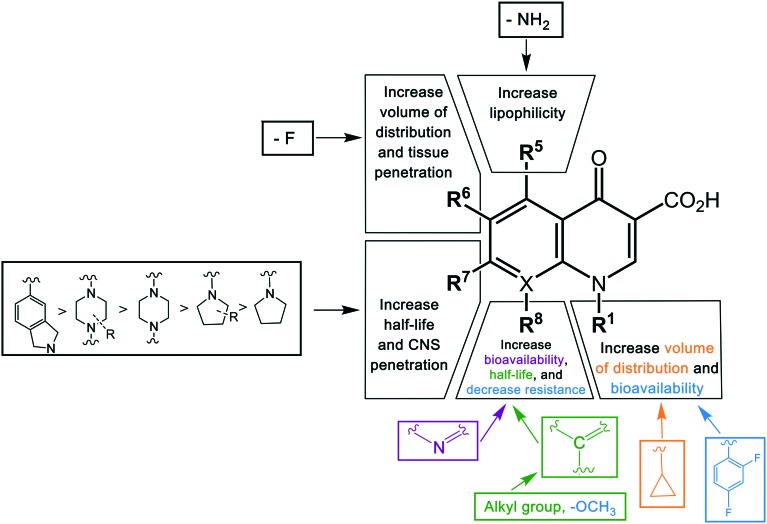
The structure–pharmacokinetic relationship of quinolones. The pharmacokinetics of quinolones is improved by modifications of different substituents in different positions. The color of the groups correlates with the color of a pharmacokinetic property.

Key structural modifications for improving the pharmacokinetics of quinolones are presented at the R_5_, R_6_, R_7_, and R_8_ positions (Fig. 3), which result in longer elimination half-life, better tissue penetration, increased volume distribution, and better bioavailability. The addition of an amino group at R_5_ increased the quinolones' lipophilicity,^67^ which can be seen from the structure of sparfloxacin. The fluorine substituent at position R_6_ proved to facilitate penetration into the bacterial cell^68^ and also improve the volume of distribution of the drug. This improvement was observed during the development of the second-generation of quinolones and was retained until the latest agent of the fourth generation, garenoxacin. The addition of substituents at the R_7_ position mediated the improvement of the half-life and bacterial tissue penetration.^5^ The azabicyclic group and piperazine group at R_7_ extended the agents' half-life to >10 h by increasing the lipophilicity.^69^,^70^ Another substituent at this position is the pyrrolidine rings; while this modification is critical for enhancing the potency of quinolones; it was reported to be associated with unfavorable water solubility and oral bioavailability.^71^ To overcome these physical properties, subsequent generations of quinolones introduced a methyl group into the rings, which can be seen from the examples of gemifloxacin and trovafloxacin.^71^ Furthermore, the alkylation of the rings at the R_7_ position increased the elimination half-life and bioavailability of the agents. The addition of a methyl group to the piperazine rings significantly increased the elimination half-life of ofloxacin, lomefloxacin, sparfloxacin, grepafloxacin, and gatifloxacin compared to enofloxacin, norfloxacin, and ciprofloxacin, which have only the piperazine group in the structure (Table 3). Alkylation at the R_8_ position was shown to increase the elimination half-life and also increase the tissue penetration of the agents.^72^ Moxifloxacin and gatifloxacin are examples of these modifications. The latest key modification is a methoxy group at this position, which lowered the development of resistance to quinolones.

### 2.3. Development in toxicological data

The most common adverse effects of the quinolones are gastrointestinal effects and arthralgia (or joint pain), which are associated with the structural feature of the quinolone pharmacophore.^30^ Due to concerns of these primary adverse effects, this class is limited for prescription to pediatric patients.^73^ In addition to these class-related toxicological disadvantages, earlier quinolones were limited in their clinical use due to several unwanted adverse effects, with some mild but frequent, and others rare but severe. Those disadvantages were reported to be dependent on the substituents in different positions on the pharmacophore and specific to particular agents (Table 4). QTc prolongation was reported to occur in patients using sparfloxacin and grepafloxacin.^5^ QTc prolongation can lead to cardiac arrhythmias.^74^ Phototoxicity was observed when using clinafloxacin and sparfloxacin.^75^ Tendon rupture, nerve damage, and fluoroquinolone-associated disability syndrome has been reported for most fluoroquinolones when they are used for a long-term period, and these side effects are proposed to be potentially permanent.^76^ Other effects include haematological toxicity with temafloxacin,^77^ hepatitis with trovafloxacin,^78^ and hypoglycaemia effects with clinafloxacin and gatifloxacin.^79^–^81^ Immunological side effects were seen in a number of agents, as were central nervous system (CNS) effects and genotoxicity (Table 4). The genotoxicity of quinolones is only seen in some fluoroquinolones when exposed to ultraviolet (UV) light, such as lomefloxacin, ciprofloxacin, and moxifloxacin. They were reported to be toxic and mutagenic following the reaction with human topoisomerase IIα in the presence of UV radiation.^156^ Some of these effects have led to quinolones being withdrawn from the market. Over time, the toxicity of quinolones has been reduced by structural modifications, and the latest agent (garenoxacin) has proved to have little adverse toxicological data.^5^ The safety profile of quinolones is being updated constantly, since some life-threatening adverse effects, such as aortic rupture and dissection caused by exposure to fluoroquinolones, have recently attracted additional warnings by the FDA in 2018. It is advised that fluoroquinolones are not to be used for patients with an aortic aneurysm, or the elderly, and only as a last-line defense.^82^

**Table 4:** The toxicological disadvantages of quinolones and the frequency observed in different agents^83^–^89^

Side effect	Agent	Frequency
Gastrointestinal effects	Sparfloxacin, grepafloxacin	>10%
	Others	2–8%
Arthralgia effects	Sprafloxacin, levofloxacin, grepafloxacin ≫ others	0.5–2%
CNS effects	Trovafloxacin	2–11% dizziness
	Levofloxacin	0.026% confusion, alteration in mentation and effect
Phototoxicity	Clinafloxacin, sparfloxacin	>10%
	Others	<2.5%
Genotoxicity	Lomefloxacin, moxifloxacin, ciprofloxacin	
QTc prolongation	Grepafloxacin, sparfloxacin	2.9%
Haematological effect	Temafloxacin	0.02% thrombocytopenia, haemolysis, and renal failure
Hepatic eosinophilia effect	Trovafloxacin	0.006%
	Grepafloxacin	12–16% transaminase elevation
	Others	<3%
Pulmonary interstitial eosinophilia	Gemifloxacin	
Immunological side effect	Gemifloxacin	
Hypoglycaemia	Clinafloxacin, gatifloxacin	
CYP 450 inhibition	Enoxacin, clinafloxacin > ciprofloxacin > lomefloxacin, ofloxacin > levofloxacin, sparfloxacin, gatifloxacin, moxifloxacin	

The substituent at the R_1_ position was shown to be related to the inhibition of cytochrome P450, with cyclopropyl and the alkyl groups at this position affected more than when substituted with a 2,4-difluorophenyl group. Other modifications leading to cytochrome P450 interactions were the replacement of the carbon atom with nitrogen at the X position, and the addition of a bulky side chain into the X_8_ of quinolones. Genotoxicity was shown to occur in agents with –NH_2_ and –CH_3_ substituents at the R_5_ position, fluorine (F) at the R_6_ and R_8_ positions, and chlorine (Cl) at the R_8_ position.^5^ Another specific structural change associated with the genotoxicity was modifications of the group at position 7, with a decrease in severe effects by pyrrolidinyl, piperazine, and alkyl groups, respectively.^30^

**Fig. 4:**
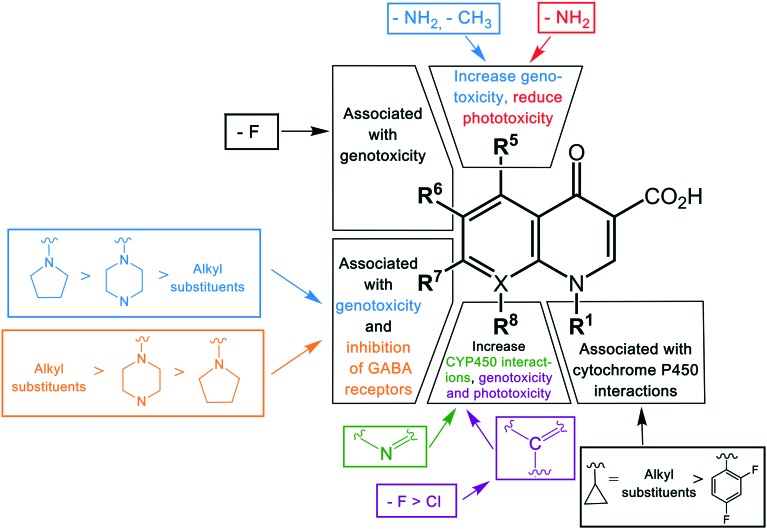
The structure–toxicity relationship of quinolones. The toxicity of quinolones is altered by modifications of different substituents in different positions. The color of the groups in the bracket correlates with the type of toxicity.

Phototoxicity is an adverse effect caused by the accumulation of susceptible drugs in the skin where they can be activated by exposure to sunlight, causing damage to the skin.^90^ This was observed in agents with an –NH_2_ group at the R_5_ position and fluorine (F) or chlorine (Cl) at the R_8_ position.^30^ Quinolones possessing this adverse effect include lomefloxacin, sparfloxacin, and clinafloxacin. Central nervous system (CNS) reactions including dizziness, insomnia, and headache have been induced by some quinolones.^91^ This adverse effect has been shown to be associated with the inhibition of GABA receptors, a major inhibitory neurotransmitter, and was observed in agents with additional groups at position R_7_.^92^ In contrast to the genotoxicity effect in this position, the degree of CNS effect increased in the reverse order, with alkyl > piperazine > pyrrolidinyl group. This highlights the difficulties in optimizing substituents against multiple parameters, with favorable changes in some properties balanced by increased detrimental outcomes in other properties. A summary of the structure–toxicity relationship of quinolones is shown in Fig. 4.

### 2.4. Conclusion

Studies of structure–activity, structure–pharmacokinetics, and structure–toxicity relations of quinolones have enabled a better understanding of different modifications to the core structure to offer the best manipulation for combining clinical efficacy, reduced toxicity, and safety. The best substituents in each position include cyclopropyl at position R_1_, fluorine at position R_6_, a pyrrolidine, piperazine, or azabicyclic group at position R_7_, and a methoxy group at position R_8_. Due to the genotoxicity of the fluoroquinolone class found to be associated with the fluorine at position R_7_, studies have been focused on the development of the fluoroquinolones. Optimizing the activity gained from other substituents (R_5_, R_1_) to offset the loss in activity by removing the fluorine has led to garenoxacin, the lead example of the fourth generation of quinolones.

### 2.5. Clinical indications and trend of quinolone use

Quinolones are antibiotics with broad-spectrum activity and a variety of clinical indications. However, the increasing rates of resistance are leading to a re-adjustment of strategies and usage for this antibiotic class,^160^ particularly to reduce the risk for selection of resistance. In recent years, fluoroquinolones have been the primary agents for treating urinary tract infections (UTIs) and infections of the digestive tract and respiratory system.^161^ Excessive prescriptions of quinolones have led to the rapid development of quinolone resistance, leading to a loss in effectiveness of this class. Australia restricted the use of quinolones in humans through a national pharmaceutical subsidy scheme and did not allow the use of quinolones in food-producing animals, leading to low rates of resistance compared to other countries.^158^ Globally, high levels of resistance to *E. coli*, the predominant cause of UTIs, has led to the replacement of quinolones by third-generation cephalosporin for this indication.^93^ Fluoroquinolones are still the mainstay for treatment of typhoid caused by *Salmonella* owing to resistance to previous first-line agents, such as ampicillin, chloramphenicol and trimethoprim/sulfamethoxazole. However, resistance to quinolones in *Salmonella* is increasingly being reported in the Americas, South Asia, Southeast Asia and especially sub-Saharan Africa, and *Salmonella* is now ranked as a high-priority pathogen for the research and development of new antibiotics.^94^,^95^ For respiratory tract infections, especially community-acquired pneumonia, quinolones are not recommended for first-line treatment but are reserved for serious cases. To reduce the development of fluoroquinolone resistance it is recommended to limit the use of this class in the treatment of patients with less severe infections, patients with prolonged hospitalized stay and patients with chronic, recurrent disease. Unfortunately, quinolones are also widely used in veterinary and husbandry practice which can lead to serious resistance through a variety of routes.^96^ High rates of resistance have been seen in food-borne pathogens such as *Campylobacter*, *E. coli*, and *Salmonella* from isolates from the United States (19%) and Spain (>80%).^97^ As mentioned earlier, resistance of these strains is much lower in Australia, where this class is not approved for animal use. This supports the argument that quinolone usage in animals increases selection of resistance.^97^

Clinical use of the fluoroquinolones is also restricted by the known and suspected toxicities of this class in specific populations, such as pregnant and breast-feeding women and pediatric and elderly patients. Although it is a powerful antibiotic for treating children's infections such as diarrhoea or Gram-negative meningitis, the toxicity (arthralgia) of this class combined with the issues of increasing resistance have reduced its use for this group.^73^ The recently reported potential for aortic rupture and dissection side effects also raises safety issues for the use of quinolones in elderly patients, where they may lead to serious bleeding or even death.^76^,^82^ In addition, there are possible teratogenic and mutagenic effects, so prescription of fluoroquinolones for pregnant and breast-feeding women is limited.^98^

Despite these increasing concerns of quinolone resistance and toxicity, the use of this class of antibiotics still remains high as it is effective for serious infections.^99^ The development of novel quinolone or quinolone-like agents with improved properties is still desirable, particularly if these new agents are only used to treat appropriate types of patients and used in an educated fashion.

## 3. Mode of action

Quinolones kill bacteria by interfering with DNA synthesis and inhibiting their replication pathway.^100^ During DNA synthesis, double-stranded DNA needs to unwind into two single-stranded structures to be used as the template, allowing the transcription complexes to proceed and complementary base pairing to occur.^101^ This unwinding process is done by the bacterial topoisomerase II type enzymes, DNA gyrase and DNA topoisomerase IV.^101^ Quinolones exert their action by inhibiting these enzymes, thereby stopping the synthesis process.

### 3.1. Quinolone target: DNA gyrase and topoisomerase IV

The two enzymes responsible for DNA synthesis in bacteria are DNA gyrase and topoisomerase IV.^101^ These enzymes take part in controlling the amount of DNA under- and over-winding, and remove the topological stress of the bacterial chromosome.^102^ Under normal conditions, DNA is highly condensed, so prior to replication it must be unwound to separate the two strands and provide the template for transcription. During the unwinding, as the replication forks move forward, super positive helical twists in the DNA are created in front of them.^101^ For the replication to proceed, the DNA topoisomerase type II enzyme removes this helical twist by cutting the DNA backbone at the double strand 4 bp apart to generate a 5′-overhang, which helps in the process of synthesizing and separating the daughter chromosomes.^102^

Although the two topoisomerase type II enzymes were reported to have similar functions and structure, there are some different physiological functions between them (Table 5). DNA gyrase uses energy in the hydrolysis of ATP to introduce negative supercoils into the DNA, resulting in the condensation of the chromosomes.^103^ In the absence of ATP, it causes relaxation of the DNA, thereby relieving the topological stress accumulated ahead of replication forks mediating the replication process.^104^ In contrast, topoisomerase IV is unable to introduce the negative supercoils. It can only relax the positive supercoils by binding to the crossovers between two interlinked daughter cells and removing knots.^104^ As a result, its primary function is associated in decatenating daughter chromosomes for the separation of two daughter cells.

**Table 5:** Function of topoisomerase type II

Topoisomerase IV	DNA gyrase
Main target in most Gram-positive bacteria	Main target in most Gram-negative bacteria
Decatenates DNA for separation into daughter cells during DNA replication	Removes positive super helical twists in the DNA ahead of replication
	Can act as topoisomerase IV in organisms that lack Top IV (such as *M. tuberculosis*, *T. pallidum*, *H. pylori*)

Genetic studies on *E. coli* strains found that the primary target of quinolone is gyrase, and the second targeted enzyme is topoisomerase IV.^105^ This is consistent with other findings that show a higher amount of gyrase-DNA cleavage complexes when incubating *E. coli* with different quinolones.^106^ On the other hand, a study by Pan and his colleague in 1996 showed that in the case of *Streptococcus pneumoniae*, the primary target of ciprofloxacin was topoisomerase.^107^ From this analysis, it was proposed that topoisomerase IV is the primary target for quinolones in Gram-positive strains, and DNA gyrase is the primary target for Gram-negative bacteria. However, several researchers have shown that this is untrue in many cases, with examples of DNA gyrase as the primary target in some Gram-positive bacteria (*e.g.* gyrase is the primary target for *Staphylococcus aureus* in treatments with norfloxacin), and conversely that topoisomerase IV is also a primary target in some Gram-negative bacteria. Moreover, subsequent studies showed that different quinolones have a different primary target for a particular strain.^109^–^111^ Therefore, investigations should be conducted on a species-by-species and drug-by-drug basis for detailed evaluation.

**Fig. 5:**
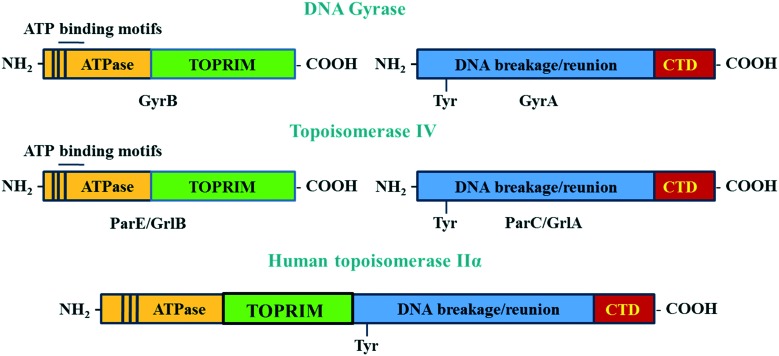
The structure of DNA gyrase and topoisomerase IV and human topoisomerase IIα. The GyrB and its equivalent domain on topoisomerase IV (ParE/GrlB) are responsible for hydrolyzing ATP during the cleavage/ligation process. The GyrA contains the tyrosine active site, which takes part in the breakage/reunion of the chromosomes. The CTD region, which is only observed in the GyrA but not in ParC/GrlA, is involved in topology recognition. Unlike two distinct domains seen in bacterial enzymes, the two subunits A and B of human topoisomerase IIα are fused together to form the homodimer enzyme.

DNA gyrase and topoisomerase IV are A_2_B_2_ heterotetramer enzymes including two pairs of identical GyrA/GyrB and ParC/ParE in Gram-negative or GlrA/GlrB in Gram-positive species.^112^ GyrA and ParC or GlrA contain an active site tyrosine residue, which is involved in the breakage/reunion of the DNA. GyrB and ParE or GlrB contain the ATPase domain and the TOPRIM domain, which are involved in the energy transduction for DNA cleavage and ligation.^113^ The differences in the physiological functions between DNA gyrase and topoisomerase IV are due to the difference in the C-terminal region of these enzymes.^113^ The C-termini of GyrA and ParC (GrlA) associating with the topological recognition are not well conserved. The addition of a CTD region in the A subunits allows DNA gyrase to generate supercoils in DNA, which cannot be modulated by topoisomerase IV^114^ (Fig. 5).

Although there is similarity in the sequence of human topoisomerase type II, IIα and IIβ with that of bacterial enzymes, quinolones have been shown to not affect the action of human enzymes. This is because the A and B subunits of human enzymes have fused during evolution, and so function as homodimers.^115^ This structure is different from the heterotetramers of bacterial enzymes. The differences in structure of bacterial and human topoisomerase II are shown in Fig. 5.

### 3.2. Quinolone action

During replication, gyrase and topoisomerase IV generate double-stranded breaks in the DNA to relax the super positive twists.^100^ This complex includes binding of the enzymes to the DNA, and is called the DNA cleavage complex. Quinolones bind to the enzyme–DNA complexes, rather than the target enzymes alone, thereby inhibiting the replication process and leading to cell death of the bacteria. There are two basic actions reported for the quinolones leading to cell death, *via* the DNA inhibition and/or *via* activation of the bacterial DNA stress response.^116^

**Fig. 6:**
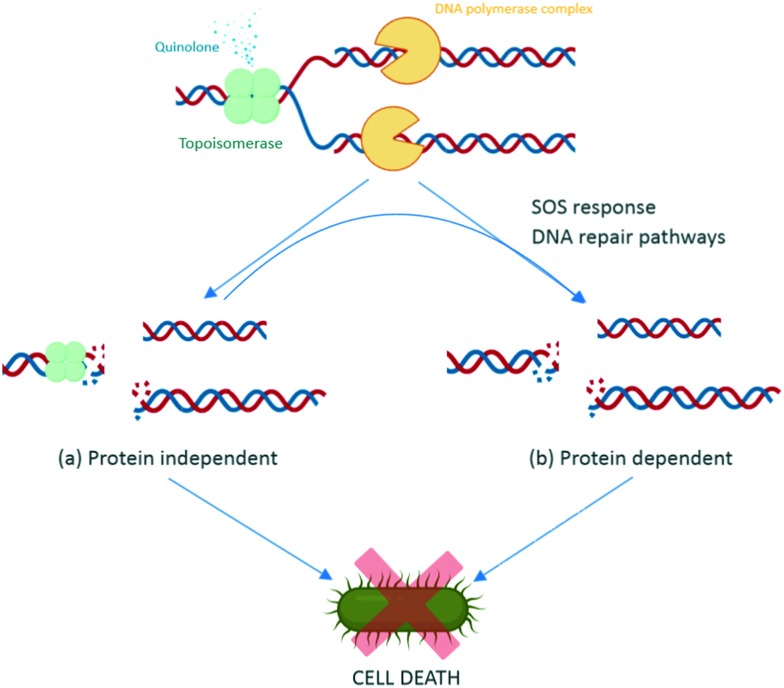
Intracellular action of quinolones. Quinolones bind to the DNA–enzyme cleavage complex at the cleavage-ligation active site. This binding creates a steady-state concentration of cleavage complexes and disrupts the replication process, which causes collision of the stabilized cleavage complexes with the DNA replication systems (replication fork, transcription complexes, and tracking systems) leading to chromosomal breaks (a). In response to this damage, SOS response and other DNA repair pathways are activated, resulting in subsequent action of the SOS system, such as extended cell filaments by expression of LexA repressor and programmed cell death by activation of toxin–antitoxin modules (b).

Quinolones bind to the DNA cleavage complex at the cleavage-ligation active site in a non-covalent manner. Two molecules of quinolones are required for this binding. The formation of the drug–DNA cleavage complex at both cleaved scissile bonds leads to the accumulation of DNA replication machinery at the replication forks.^117^ Due to their intercalation, quinolones strengthen the stable state of the cleavage complex by acting as a physical block to the ligation, resulting in bacteriostasis with low concentrations of quinolone and bactericidal activity with lethal concentrations.^118^ Moreover, when the DNA tracking systems collide with these drug–DNA cleavage complexes, permanent chromosome breaks are generated, triggering the DNA stress response.^157^ This activates RecA protein and promotes self-cleavage of the LexA repressor, thus de-repressing the expression of SOS response genes.^119^ Therefore, preventing the induction of SOS response leads to an inability to repair DNA breakage. The increase in the DNA breakages combines with the disabled SOS system to augment the bactericidal potency of quinolones (Fig. 6).^119^ Some studies have reported that reducing the number of targeted enzymes reduces the activity of quinolones. Moreover, the results from a study on the contribution of the reactive oxygen species to the quinolone-mediated bactericidal action showed that death was mainly a protein synthesis-dependent mechanism.^120^ The bactericidal activity of quinolones is potentially due to both the inhibition of DNA synthesis and the subsequent bacterial response through stress-induced protein expression. However, while the primary target of quinolone antibiotics is clear, the underlying molecular mechanism of activity leading to death is still unclear, as with many classes of antibiotics.

Another action of quinolones is inhibiting the activity of the bacterial topoisomerase II. Following inhibition of the ligation of the enzymes, they also disrupt the catalytic functions of the enzymes. Therefore, this adds to the overall toxicity of the quinolones by acting as a catalytic inhibitor.

### 3.3. Enzyme–quinolone interactions

**Fig. 7:**
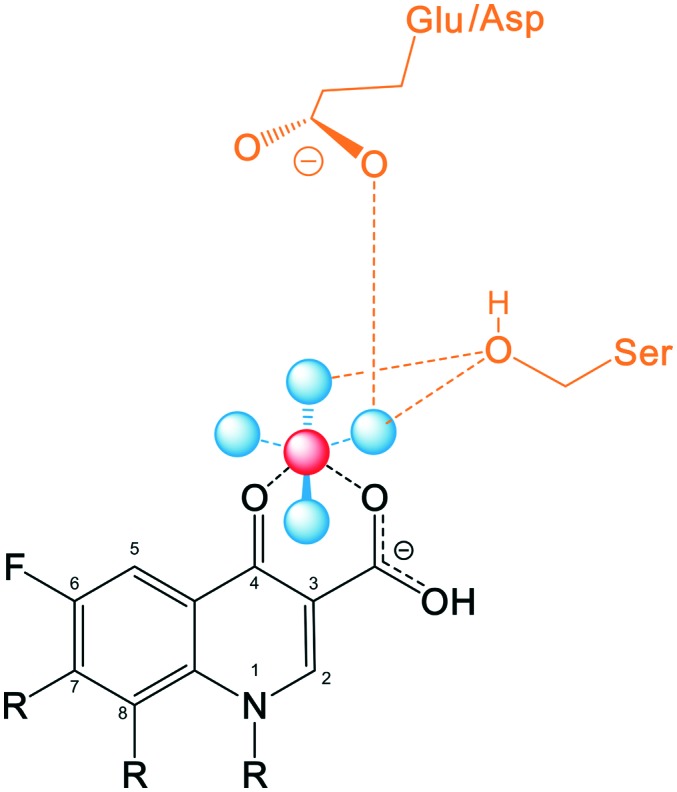
A simplified diagram of the water–metal ion bridge between a fluoroquinolone and topoisomerase IV – DNA cleavage complex. Fluoroquinolone (black) binds *via* a non-catalytic Mg^2+^ ion (red) through four water molecules (blue) that fill out the coordination sphere of the Mg^2+^ ion, interacting with the side chains of the serine and acidic residues (yellow).

Recent studies including structural and functional analysis studies have shown that the binding of quinolones to bacterial topoisomerase type II enzymes is *via* a water–metal ion bridge.^121^ This interaction is mediated by a noncatalytic Mg^2+^ ion coordinated with four water molecules, forming a bridge for hydrogen bonds between the bound quinolone and the active site serine and acidic residues. The interaction sites on the quinolones are located on the R_3_/R_4_ keto acid of the drug pharmacophore (Fig. 7), supporting the tolerance for structural development with substituents at positions R_1_, R_7_, and R_8_ that lie on the opposite side of the molecule from this position. In contrast, alterations at R_5_, R_6_, and R_2_ are likely to disrupt this interaction due to their proximity.

This structure also explains why the quinolones do not interfere with the human topoisomerase type II enzymes, mediating the safety of this class of drug, as the human enzyme does not have the serine and acidic residues which are essential for anchoring the water–metal ion bridge.^122^

## 4. Mode of resistance

The emergence of resistance to the quinolones is becoming a critical issue that is limiting the use of this class of antibiotics. Mechanisms of resistance are classified into two different categories, based on mutations in the bacteria genes (mutation in genes encoding the DNA gyrase and topoisomerase IV targets, or other genomic alterations that affect antibiotic accumulation) *versus* acquisition of resistance genes from other sources (plasmid acquisition from the environment or other resistant strains, with multiple pathways of resistance).

### 4.1. Mutation

#### 4.1.1. Change in genes encoding the targeted enzymes

As discussed in the section on mode of action, DNA gyrase and topoisomerase IV are the two targeted enzymes of quinolones. Mutation in single amino acids in either one of these two enzymes weakens the interaction between the quinolones and enzymes, reducing quinolone susceptibility. These mutations have been reported to be primarily located on the amino terminal domains of GyrA or ParC of the enzymes (Table 6). The most common mutated amino acids are the serine residue and an acidic residue (glutamic acid or aspartic acid) four amino acids away.^123^ Mutations of Ser83 and Asp87 are the most common resistance mutations in GyrA of *E. coli*, with similar mutations in other species at the equivalent positions.^124^ This domain of the enzymes has been shown to be responsible for anchoring the water–metal ion bridge, which is termed as the quinolone resistance-determining region (QRDR). Mutations at this QRDR disrupt the water–metal ion bridge, thereby reducing drug affinity for the enzyme–DNA complex. Mutation at the serine accounts for more than 90% of the mutant pool, followed by the mutations at the acidic residue.^125^,^126^ Mutations at the serine residue on GyrA and ParC appear to have little effect on the catalytic activity of the enzymes. However, the mutations at the acidic residue were reported to significantly reduce the catalytic activity from 5- to 10-fold.^115^ Presumably this explains why mutation occurs more often at the serine residue, as it does not impact enzyme activity. It is notable that the serine residue is highly conserved across bacterial species despite its minimal contribution to the activity of the enzymes. Based on a study on nybomycin on *Streptomyces* spp., it was proposed that this conserved serine residue is responsible for protection against ‘natural’ antibiotics rather than synthetic antibiotics.^127^ Mutations in the amino acids of the GyrB and ParE domains also cause quinolone resistance; however, they are less frequent than the mutations located on the GyrA and ParC. Based on the X-ray crystallography analysis of the structure, it was reported that the QRDRs of the GyrB/ParE are distant from the QRDRs of the GyrA/ParC. However, the structure also showed that the conformation of these two QRDRs is homologous to each other. Other structural studies on co-crystals of the quinolones and these domains have shown that the mechanism of resistance in these domains is similar to that in the GyrA/ParC domains *via* mutations of the QRDRs by charge interactions to decrease the drug affinity.

**Table 6:** The mutations detected in DNA gyrase and topoisomerase IV genes^129^–^138^

Species	gyrA	gyrB	parC	parE
*E. coli*	Tyr50Phe	Asp426Asn	Ala56Thr	Leu416Phe
	Ala51Val	Lys447Glu	Ser57Thr	Ile444Phe
	Ala67Ser	Ser429Asn	Asp69Glu	Leu445His/Ile
	Gly87Cys		Gly78Asp	Ser458Ala/Pro/Thr/Trp
	Ser80Arg/Ile		Ser80Arg/Ile	Glu460Asp/Lys
	Gly81Asp/Cys		Ser83Leu	Ile464Phe
	Asp82Gly		Glu84Ala/Gly/Lys/Val	Ile529Leu
	Ser83Ala/Ile/Leu/Trp/Tyr/Val		Cys107Trp	
	Ala84Pro/Val			
	Asp87Asn/Glu/Gly/His/Tyr/Val		Ala108Thr/Val	
	Gln106Arg/His			
	Ala119Glu			
	Ala196Glu			
	Arg237His			
*Salmonella* spp.	Ala67Pro	Tyr420Cys	Glu21Lys	Glu453Gly
	Asp72Gly	Gly434Leu	Thr57Ser	Ser458Pro
	Val73Ile	Gly435Ala/Glu/Val	Thr66Ile	Glu459Thr
	Gly81Asp/Gly	Arg437Leu	Gly72Cys	His461Tyr
	Ser83Ala/Leu/Phe/Thr/Tyr	Gly447Cys	Gly78Asp	Gly468Cys
	Asp87Asn/Gly/Lys/Tyr	Ser464Phe/Tyr	Ser80Arg/Ile	Ser493Phe
	Leu98Val	Glu466Asp	Glu84Gly/Lys	Ala498Thr
	Ala119Glu/Ser/Val	Ala468Glu	Phe115Ser	Arg507Ile
	Ala131Gly	Leu470Met	Ala141Ser	Val512Gly
	Glu133Gly			Lys514Asn
	Glu139Ala			
*Proteus mirabilis*	Ser83Arg/Ile	Ser464Phe/Tyr	Gly78Asp	—
	Glu87Lys	Glu466/Asp	Ser80Arg/Ile	
			Addition of lysine between K455 and A456	
*Capnocytophaga* spp.	Gly80Asn/Asp	—	Met55Ile	Gly377Asp
	Asp81Gly		Glu101Gln	Lys410Gln
	Ser82Phe/Tyr			Ile502Xaa
	Thr82Ile			Thr503Xaa
	Asp86Tyr			Phe504Xle
				Phe508Xaa
				Phe509Xaa
				GLu511Xaa
				Glu515Asp
*Clostridium perfringens*	Gly81Cys	Ala431Ser	Asp11Tyr	V637
	Asp82Asn	172V	Val22Phe	Glu486Lys
	Ser83Leu		Asp88Tyr	
	Asp87Tyr		Ser89Ile	
	Ala119Glu		Asp93Tyr	
			Ala131Ser	
			Val196Phe	
			Asp502Tyr	
*S. aureus*	Ser84Ala/Leu	Val28Ala	Ile45Met	Gly78Ser
	Ser85Pro	Ile56Ser	Ser80Phe/Tyr	Gly107Ser
	Glu88Lys/Gly	Gln66Lys	Glu84Lys	Arg136Gly
	Val248Glu	GLy85Ser	Pro144Ser	Asn139Ser
	Gly255Arg	Asp89Gly	Ile233Val	Ser230Gly
	Ala457Thr	Ile102Ser/Thr	Ser267GLy	Val327Ile
	Asp483Glu	Ser128Leu	Arg372His	HLu422Asp
	Asp495Asn	Arg144Ser/Ile	Arg400Cys	His478Tyr
	Glu594Gly	Thr173Ala	Glu404Gly	Gly530Asp
	Val598Ile	Glu317Asp	Tyr410Phe	Glu596Asp
	Ser668Ala	Asp437Asn	Phe521Tyr	Val609Leu
	Val712Ile	Arg458Gln	Phe594Tyr	
	Thr818Val	Gly491Asp	Asp641Asn	
	Arg837His	Glu568Lys	Lys650Arg	
	Asp856Glu		Val656Ile	
	Asn860Thr		Ala688Val	
	Glu886Asp		Met694Val	
*S. pneumoniae*	Ala17Thr	Val432Asp	Ser52Gly	Asp435Asn
	Gly54Val	Asp435Asn/Glu/Ile	Gly77Glu	Pro454Ser
	Val71Ile	Glu474Lys	Asp78Asn	Ile460Val
	Asp80Ala		Ser79Phe/Tyr	Glu474Lys
	Ser81Phe/Tyr		Asp83Ala/Asn/Gly/Tyr	
	Ser83Phe/Tyr		Asn91Asp	
	Glu85Gly/Lys		Gly128Asp	
	Glu87Lys/Gln		Gly135Asp	
	Trp93Ser		Lys137Asn/Asp	
			Ala142Ser	

The degree of resistance caused by mutation of a single amino acid in the enzymes varies among bacterial species and quinolones. Different bacterial strains have different primary targets for quinolones, thereby having different relative sensitivities to a given quinolone. Resistant clinical isolates indicate that a single target-site gene mutation on either of the two enzymes results in an 8–16-fold increase in resistance.^126^ A single target-site mutation in both DNA gyrase and topoisomerase IV results in increasing the levels of resistance. Sequential mutations in both target enzymes in clinical isolates increases the resistance up to 100-fold.^128^ Some bacteria, such as *Mycobacterium tuberculosis*, *Treponema pallidum*, and *Helicobacter pylori*, have only the DNA gyrase enzyme.

#### 4.1.2. Other genomic mutations

As DNA gyrase and topoisomerase are cytoplasmic enzymes, quinolones must pass through the bacterial envelope to exert their functions. Therefore, quinolone activity is also affected by their ability to penetrate the cellular barrier and the effectiveness of efflux pumps at removing the antibiotic from the cytoplasm. Quinolones are known to enter bacterial cells by using both porin- and lipid-mediated pathways. Therefore, resistance can occur *via* mutations that reduce drug accumulation by under-expression of porins, by over-expression of efflux pumps, or by modifications of the lipopolysaccharide (LPS) structures.

Many quinolone-resistant strains do not have mutations in the enzymatic target QRDR^139^ and were less susceptible to unrelated compounds, such as cyclohexane, salicylate, and tetracycline, proving that resistance is associated with broad-spectrum efflux activity.^140^ The multiple antibiotic resistance (*mar*) gene is known to cause tolerance to a variety of compounds.^141^ Mutation of this gene leads to both over-expression of the acrAB efflux pump and reduced expression of OmpF (outer membrane protein F) porin. MarA, a positive regulator of acrAB transcription, can be induced either by mutation of the mppA gene or by exposure to salicylate and tetracycline. Thereby, salicylate and tetracycline may stimulate quinolone resistance. Moreover, MarA prevents translation of OmpF and activates the expression of OmpX, which is a porin expression down-regulator, thus reducing the expression of a variety of porins, such as OmpC, OmpD, OmpD, OmpF, LamB, and Tsx.^142^,^143^

Another gene that contributes to the resistance against quinolones and other antibacterial agents is the *nfxB* gene, which confers alterations in expression of functional OmpF at the cell surface OmpF, thereby decreasing quinolone entry.^144^ In addition, modifications of OmpA, a β-barrel protein associated with the integrity of the cell envelope or acting as a porin, depending on the species, may lead to reduced quinolone susceptibility,^4^ as can changes in SoxRS regulons resulting from bacterial adaption to superoxide stress.^140^

Quinolone resistance associated with efflux pumps include modification of the major facilitator superfamily (MFS) of Gram-positive bacteria or the resistance–nodulation–division (RND) family, multiple antibiotic and toxin extrusion (MATE), and ATP-binding cassette (ABC) of Gram-negative bacteria.^142^ Mutations of efflux systems can alter their specificity for quinolones or cause upregulation.

### 4.2. Acquisition of resistance plasmids

In addition to resistance caused by mutations in the bacterial genome, quinolone resistance also occurs *via* plasmid-mediated mechanisms. Plasmids carrying the quinolone resistance genes can cause serious clinical issues, with 10–250-fold decreases in susceptibility.^145^ The transmission of these resistance plasmids is through horizontal transfer from bacteria to bacteria as well as vertical transfer from generation to generation. Three reported gene families are involved in this plasmid-mediated quinolone resistance (Table 7). They reduce the bacterial susceptibility to quinolones and mediate the selection of mutants promoting treatment failure.

**Table 7:** The plasmid-mediated quinolone resistance gene

*qnr* gene	
*qnrS*	DNA mimics
*qnrB*	Decreases binding of enzymes to DNA → lowering the enzyme targets on the chromosome
*qnrC*	
*qnrD*	Binds to the enzymes and inhibits the quinolones from entering the cleavage complexes
*qnrVC*	
*aac(6′)-Ib-cr* include 2 mutations	
Trp102Arg	Variant of aminoglycoside acetyltransferase
Asp179Tyr	Acetylates the unsubstituted N of the C_7_ piperazine ring → decreases drug activity
Plasmid-mediated quinolone efflux pumps	
OqxAB	Increases efflux pump activity
QepA	Decreases susceptibility to hydrophilic quinolones

The first plasmid-encoded protein is Qnr, a pentapeptide repeat family protein.^146^ These proteins are folded into a right-hand quadrilateral β-helix shape and dimerize to format rod-like structure with a size, shape, and electrostatic surface mimicking that of β-form DNA.^147^ More than 100 variants have been discovered in clinical isolates, which are classified into 6 subfamilies (*qnrA*, *qnrB*, *qnrC*, *qnrD*, *qnrS*, and *qnrVC*).^146^ The qnr gene has been reported to originate from the chromosomes of many aquatic bacteria; with *qnrA* originally from *Shewanella algae*, *qnrB* from *Citrobacter* spp., *qnrC*, *qnrS*, and *qnrVC* from *Vibrio* spp., and *qnrD* and *qnrE* from *Enterobacter* spp.^148^ These Qnr proteins compete with DNA binding to the enzymes, thereby inhibiting the quinolone from entering the cleavage complexes and reducing the number of double-stranded breaks on the chromosomes, resulting in reduced quinolone toxicity to the chromosomes.

The second plasmid-encoded protein involved in quinolone resistance is AAC(6′)-Ib-cr, a derivative of aminoglycoside acetyltransferase that has Trp102Arg and Asp179Tyr mutations.^149^ These two unique mutations distinguish this variant enzyme from other ACC(6′)-Ib enzymes, and leads to specific targeting of quinolones with an amine on the piperazinyl ring skeleton, such as ciprofloxacin, norfloxacin, and enoxacin. The enzyme acetylated the unsubstituted nitrogen of the R_7_ piperazine ring, thereby decreasing the quinolone activity. Both mutations are necessary for this specific enzyme action, with the Trp102Arg mutation positioning the Asp179Tyr tyrosine aromatic ring for optimal interaction with the quinolone, anchoring it in place.^149^

The third family associated with quinolone resistance is the plasmid-mediated quinolone efflux pumps including OqxAB and QepA. OqxAB is a multidrug-resistant efflux pump encoded by conjugative plasmid pOLA52 found in *E. coli* strains isolated from swine manure. It was recently detected in human clinical isolates of *E. coli* and *K. pneumoniae*. Bacteria with this oqxAB gene were 8- to 16-fold less susceptible to nalidixic acid and ciprofloxacin, respectively. This efflux pump not only mediates low-level quinolone resistance but also helps bacteria to survive under low concentration of quinolones, thus facilitating the subsequent development of higher level resistance.^149^ Another novel plasmid-mediated quinolone efflux pump is QepA, which is encoded by pHPA plasmid found in clinical isolates of *E. coli* from Japan. It is an efflux pump of the major facilitator family that decreases bacterial susceptibility to hydrophilic quinolones.^11^ These multidrug-resistant efflux pump encoded genes do not directly cause high levels of resistance to quinolones, but can facilitate the development of mutations to topoisomerase enzymes by allowing the bacteria to adapt to low concentrations in quinolones inside the bacteria.

## 5. Future development of quinolone antibiotics

**Fig. 8:**
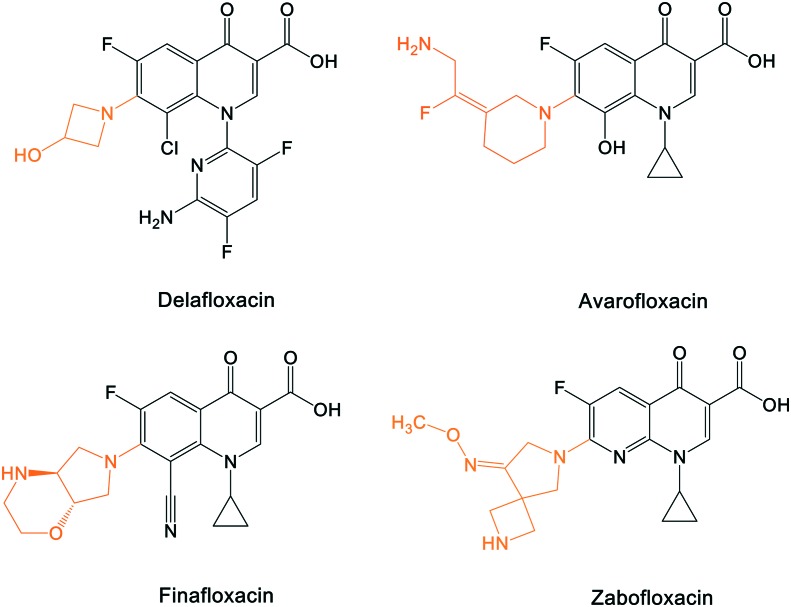
The structure of novel quinolones and their C_7_ substituents. The varied C_7_ substituents are presented in orange.

A number of other quinolones have recently been approved or are under advanced clinical development (Table 8).^150^–^152^ The C_7_ substituents of classical quinolones lack a strong basic group, so the quinolones have weak acidity. Basicity can enhance their activity in acidic environments, including phagolysosomes, inflammatory cells related to infection sites such as skin, soft tissue, vagina, and urinary tract. Studies by Kocsis and his colleagues have reviewed four potential substituents at the R_7_ position that could increase the activity of the compounds in an acidic environment including 3-hydroxy-1-azetidinyl (delafloxacin, FDA approved in June 2017), (3*E*)-3-(2-amino-1-fluoroethylidene)piperidinyl (avarofloxacin, ceased development after phase 3), pyrrolo-oxazinyl (finafloxacin, FDA approved 2014), and (8*E*)-8-methoxyimino-2,6-diazaspiro[3.4]octan (zabofloxacin, phase 3)^153^ (Fig. 8).

**Table 8:** The quinolone pipeline

Compound	Development phase	Developer	Chemical structure
Delafloxacin	Approved	Melinta (*via* Wakunaga Pharmaceutical and Rib-X Pharmaceutical)	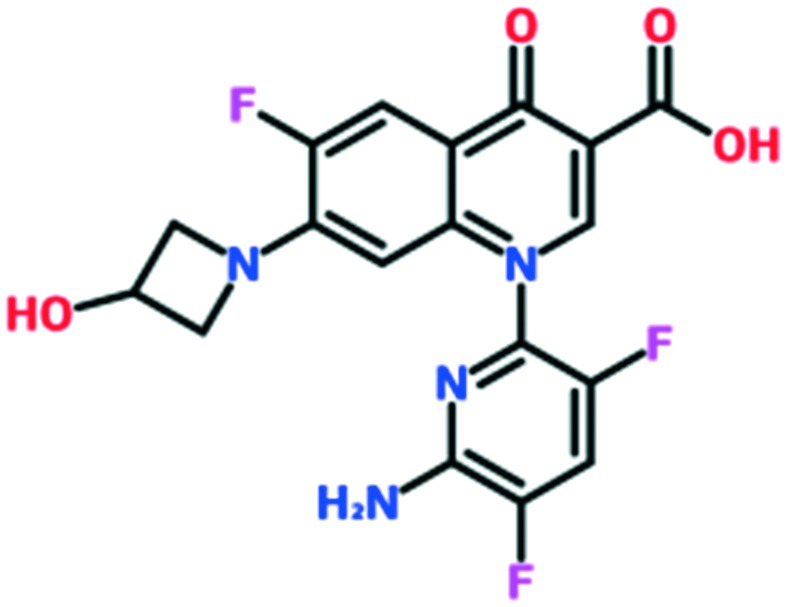
Finafloxacin	Approved for otic suspension	MerLion Pharmaceuticals Pte Ltd.	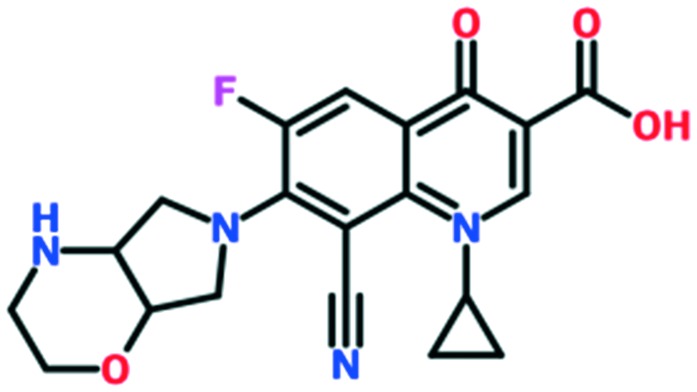
Ozenoxacin	Approved in Japan (2015), topical cream approved in USA/Canada for impetigo	Maruho Co (*via* Toyama Chemical) in Japan, Ferrer Internacional in Europe, Medimetriks in USA	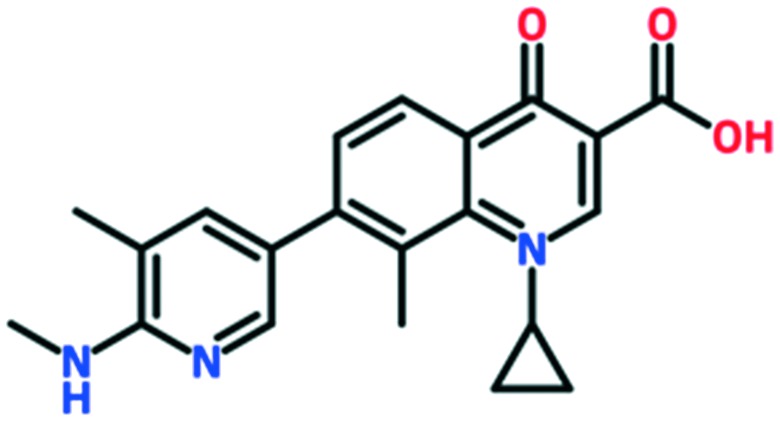
Avarofloxacin	Completed phase 3, development halted	Furiex Pharmaceuticals	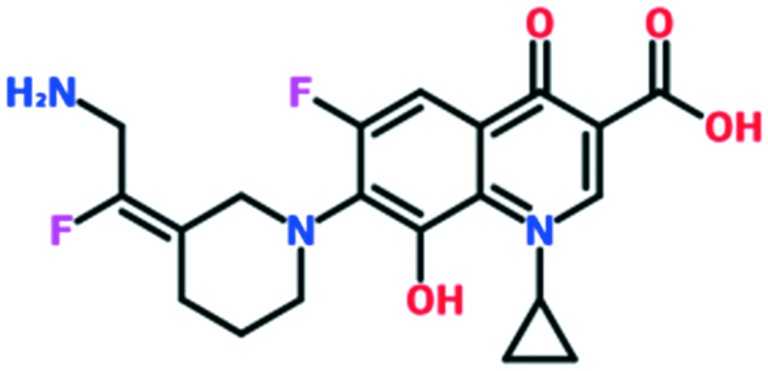
Cadazolid	Completed phase 3, development halted 2018	Johnson & Johnson (*via* Actelion)	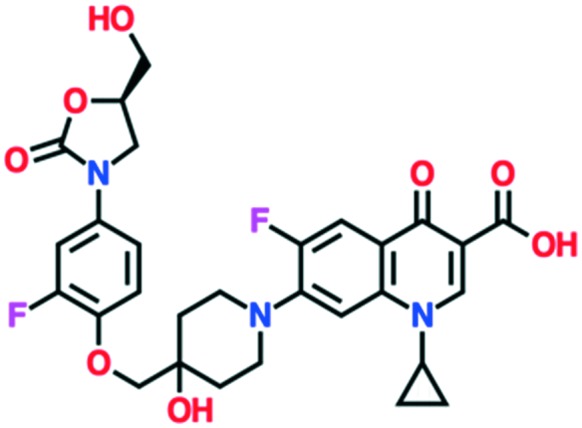
Zabofloxacin	Phase 3	Dong Wha Pharmaceuticals/Pacific Beach BioSciences	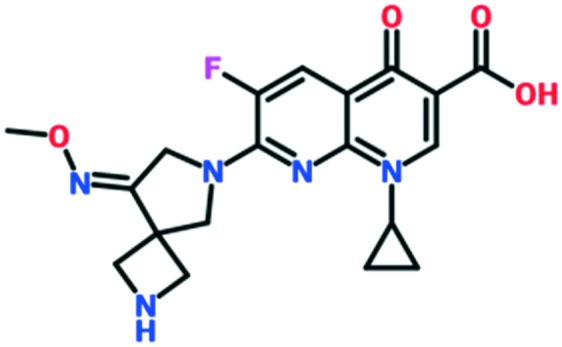
Lascufloxacin	Phase 3 completed	Kyorin Pharmaceutical Co. Ltd.	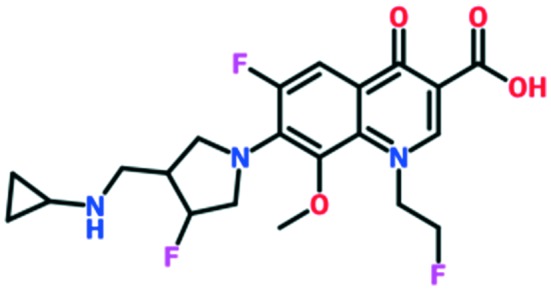
Nemonoxacin	Phase 2 (marketed in Russia, Taiwan, China, as Taigexyn)	TaiGen Biotechnology Co. Ltd.	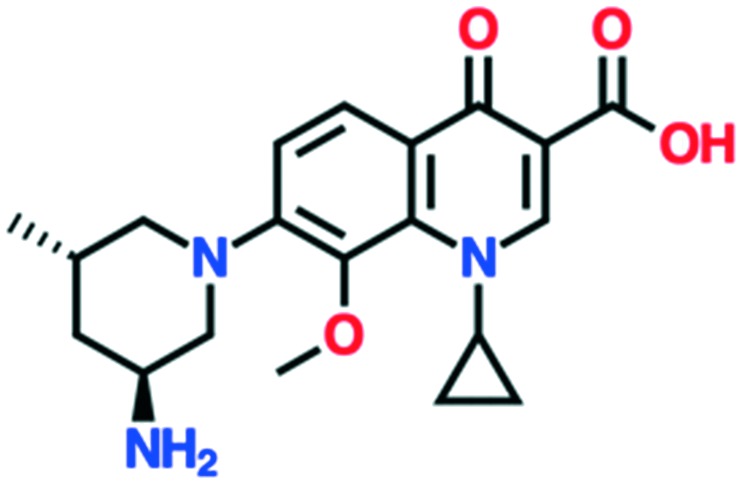
OPS-2071	Phase 2	Otsuka Pharmaceutical Co. Ltd.	Unknown
Levonadifloxacin (WCK 771) + alalevonadifloxacin (WCK 23491, oral prodrug)	Phase 2	Wockhardt Ltd.	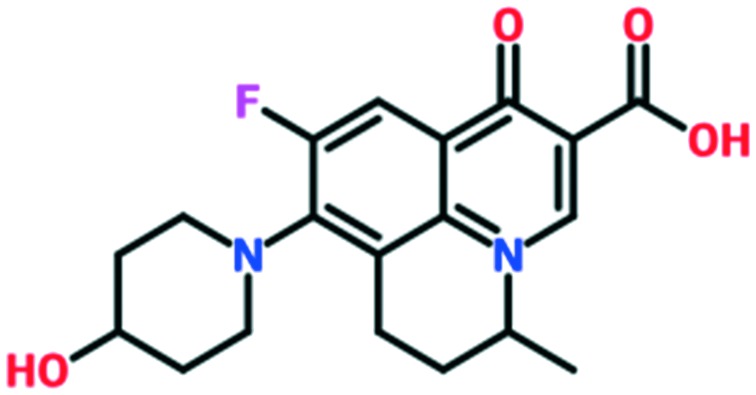
TNP-2092 (a rifamycin–quinolizinone hybrid)	Phase 1	TenNor Therapeutics	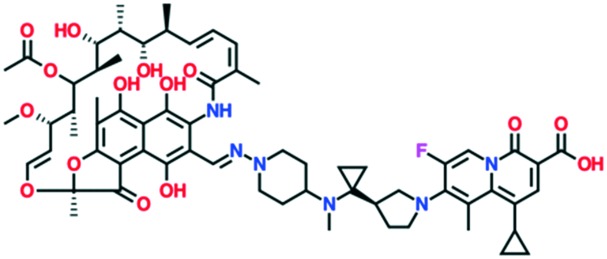
MCB3837 (oxazolidinone–quinolone hybrid)	Phase 1	Deinove SA (formerly Morphochem AG)	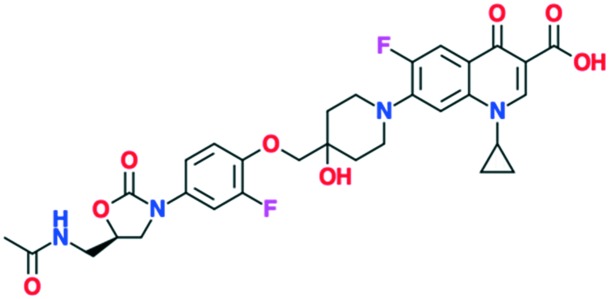

These novel quinolones have been found to result in improved potency and safety profile and decreased toxicity compared with classical quinolones.

A number of other quinolones are in various stages of development (Table 8).^150^–^152^ Several of these are hybrid antibiotics, in which the quinolone scaffold is attached to either rifamycin (TNP-2092) or an oxazolidinone (MCB3837) to increase the spectrum of activity. A related fluoroquinolone–oxazolidinone hybrid, cadazolid, was developed by Actelion and progressed through phase III trials for *Clostridium difficile* infections, but after Actelion was acquired by Johnson & Johnson in June 2017 further development was discontinued in April 2018 after meeting the endpoint in only one of two trials.

**Fig. 9:**
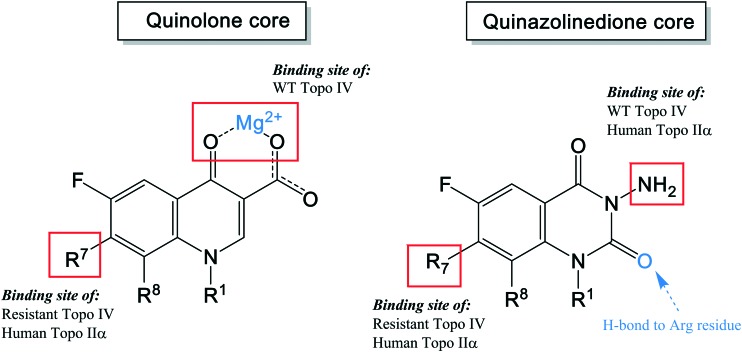
The pharmacophore structure of quinolones and quinazolinediones showing different potential binding sites.

As shown in the description of quinolone resistance, the main resistance mutations are located on the DNA gyrase and topoisomerase IV enzymes, which disrupt the quinolones from the cleavage complexes. Therefore, several researchers have focused on developing novel agents that could overcome this resistance. While many attempts have been made, the most recent studies have attempted to modify the structure to find a new binding site on the enzymes, distinct from the water–metal ion bridge. Based on this approach, quinazolinediones have been proposed as a new class of antimicrobial compound. They possess a similar structure to quinolones but do not contain the keto acid moiety that associates to the water–metal ion bridge interactions^154^ (Fig. 7). The keto acid is replaced by an R_2_ carbonyl that can bind to the enzyme's conserved arginine residue by a hydrogen bond (Fig. 9).^108^ This binding seems to overcome the resistance generated against quinolones; but the hydrogen bond interaction was shown to be weaker than the metal-ion interaction of quinolones. However, QnrA has been reported to reduce susceptibility to quinazoline-2,4-diones.^159^ Other studies on quinazolinediones have demonstrated that some agents in the quinazolinedione class with a 3′-(aminomethyl)pyrrolidinyl as R_7_ substituent produce stronger binding to the bacterial topoisomerase type II enzymes.^155^ However, this substituent also targets the human type IIα enzymes, leading to toxicity and therefore cannot be used for human therapy. Future studies should invest in identifying novel C_7_ substituents for quinazolinediones that are selective for bacterial over human enzymes. It is possible that the types of basic C_7_ substituents found in the new fluoroquinolones recently approved or under development could be applied to the quinazolinedione scaffold (Fig. 9).

## 6. Conclusions

Quinolones are a class of synthetic bactericidal antibiotics with broad-spectrum activity, which can inhibit both Gram-negative and Gram-positive bacteria, including anaerobes. They exert their activity by binding to the bacterial topoisomerase type II enzymes, interfering with the DNA synthesis pathway. Binding to the cleavage complex occurs *via* a water–metal ion bridge, which links the keto carbonyl group of quinolone indirectly to the serine and acidic residue of the enzymes mediated by a Mg^2+^ ion.

As with other antibiotics, this class is faced with a rapid increase in global levels of resistance, either through self-generating mutations or *via* plasmid-mediated acquisition. The main genomic mutations occur by altering the enzyme target active site serine residue, which accounts for more than 90% of the mutant pool. Quinazolinediones, a related structural class of antibiotics, do not rely on this critical interaction for their binding to the same enzyme, and so have potential for further development if potency can be improved and human enzyme interactions reduced.

The quinolones can be modified at the R_1_, R_6_, R_7_, and R_8_ (and, less commonly, R_5_) positions to optimize activity, pharmacokinetics, and toxicity. The best substituents at each position are a cyclopropyl group at R_1_, a fluorine at R_6_, an azabicyclic group at R_7_, and a methoxy group at R_8_. Although the fluorine at position R_6_ significantly improves quinolone activity, current research is focused on its removal because it is related to genotoxicity. The reduction in potency on its removal can be compensated for by using alternative substituents at R_5_ (amine, –NH_2_) and R_1_ (cyclopropyl or 2,4-difluorophenyl), which can give the same potency as the fluorine at R_6_ but with reduced toxicity.

It seems clear that improvements in activity and anti-resistant properties are still possible, and new generations of quinolones can still contribute to the effective treatment of bacterial infections, as reflected by the number of new analogues in the clinical pipeline.

## Conflicts of interest

There are no conflicts to declare.
